# Comparative study of endolaser versus cryocoagulation in vitrectomy for rhegmatogenous retinal detachment

**DOI:** 10.1186/s12886-019-1099-9

**Published:** 2019-04-25

**Authors:** Maico Bentivoglio, Christophe Valmaggia, Hendrik P. N. Scholl, Josef Guber

**Affiliations:** 1 Eye Clinic, Cantonal Hospital Sankt Gallen, Rorschacherstrasse 95, CH-9007 Sankt Gallen, Switzerland; 2 Institute of Molecular and Clinical Ophthalmology Basel (IOB), Basel, Switzerland; 3 Department of Ophthalmology, University of Basel, Basel, Switzerland; 4 Wilmer Eye Institute, Johns Hopkins University, Baltimore, USA

**Keywords:** Retinal detachment, Endolaser, Cryocoagulation, Retinopexy, Pars plana vitrectomy, Rhegmatogenous retinal detachment, Surgical outcome, Risk factors

## Abstract

**Background:**

To investigate the influence of different types of retinopexy on the outcome of rhegmatogenous retinal detachment (RRD) repair.

**Method:**

All patients with RRD who underwent pars plana vitrectomy (PPV) between January 2013 and December 2017 were included. Analysed surgical factors were types of retinopexy (cryocoagulation, endolaser, combined). Subgroup analysis was performed in patients with primary proliferative vitreoretinopathy (PVR), and/or the necessity of a primary silicone oil fill.

**Results:**

A total of 1017 eyes with retinal detachment were included. The predominant type of retinopexy used during PPV was cryocoagulation in 492 eyes, followed by a combined cryocogulation/endolaser in 306 eyes and laserretinopexy in only 219 eyes. Overall, the re-detachment rate was 10.1%. In most of the cases (53.6%) the main reason for re-detachment was insufficient retinopexy, followed by a PVR-reaction in 37.3%, and new site break in 9.1%. No significant difference in the rate of re-detachment was found between the different types of retinopexy (*p* = 0.309). However, subgroup analysis showed a significantly higher rate of re-detachment in patients with a primary PVR (*p* = 0.0003), and in the group with silicone oil as the primary tamponade (*p* = 0.0001).

**Conclusion:**

The data suggests that the type of retinopexy has little relevance for the surgical outcome of PPV for the primary RRD. However, patients with primary PVR and primary silicone oil fills were at a significantly increased risk for re-detachment.

## Background

Only a hundred years ago, rhegmatogenous retinal detachment (RRD) was declared untreatable at the International Congress in Paris. Since then, a few new methods have been developed [1]. Pars plana vitrectomy (PPV), which was first introduced in 1972 by Machemer, has revolutionized the treatment of RRD. Over the last few years, PPV has undergone a major technical development towards small incision vitreoretinal surgery, and has become a standard procedure for almost all types of retinal detachments [2, 3].

An integral part of the treatment of all kinds of RRD is to create a barrier around the retinal break [4, 5]. Usually, this is performed by using either cryocoagulation or endolaser. Both techniques lead through scarring to create a durable chorioretinal connection after 2–3 weeks [6–8]. Another crucial step in the treatment of RRD is the use of a tamponade such as gas or silicone oil until the barrier around the retinal break becomes stable [4, 6]. To date, there is limited evidence as to which type of retinopexy provides the best outcomes. However, some older studies have shown that cryotherapy can be a stimulating factor for postoperative proliferative vitreoretinopathy (PVR) [9–12].

This present study compares the retinopexy techniques regarding the surgical failure rate after PPV for rhegmatogenous retinal detachment.

## Method

The study was approved by the local ethic committee(Ethikkommission Ostschweiz, Business Administration System for Ethics Committees number 2018–00104) and was performed as a part of departmental quality control. Research adhered to the tenets of the Declaration of Helsinki.

All patients with RRD who underwent PPV at the Cantonal Hospital Sankt Gallen (tertiary referral centre) between January 2013 and December 2017 were included. Patients with other types of retinal detachment, such as tractional or exsudative types, were excluded. Collected data included: demographics, side, macula involvement, PVR preoperatively, lens status (phakic, pseudophakic), type of tamponade (oil, gas), occurrence and reason for re-detachment (PVR, insufficient retinopexy, new break).

All surgeries were performed by three experienced vitreoretinal surgeons. The type of retinopexy was determined during the procedure, and was solely chosen according the surgeon’s preference. In phakic eyes, phacoemulsification and PPV were both performed in one procedure.

All patients were followed up for six months in our outpatient clinic. Thereafter, patients were referred back to the private ophthalmologist. In case of re-detachment after six months, patients were referred back to hospital for re-treatment.

### Surgical technique

A standard core and peripheral 3-port PPV (23-gauge) (Stellaris, Bausch and Lomb, New York, USA) was performed for all patients. Surgery was performed under general anaesthesia. After a complete vitrectomy and the separation of posterior hyaloids with suction techniques, retina reattachment was obtained either by direct fluid-air exchange with the drainage of subretinal fluid through the main break, or by using perfluorocarbon liquid followed by fluid-air exchange. Adjuvant retinotomies were performed to achieve complete retinal reattachment, if required. Retinopexy was performed either, with transconjuctival cryocoagulation or by endolaser. The type of retinopexy was determined during the procedure and was solely chosen according the surgeon’s preference. In some eyes, both retinopexy types were applied, e.g. if additionally to a retinal break, large lattice area or if multiple breaks were present. At the end of surgery, gas (20% Sulfur hexafluoride [SF_6_ gas], 12% perfluoropropane [C_3_F_8_ gas]) or silicone oil tamponade was applied. The choice of intraocular tamponade depended on RRD characteristics. However, as a rule, SF_6_ gas was injected in case of retinal breaks located in the upper 240 retinal degrees; while C_3_F_8_ gas was injected when inferior retinal tears were present and in patients with low-compliance posture. Silicone oil tamponade were reserved for complex RRD’s, such as PVR and giant retinal tears. In phakic eyes, phacoemulsification and PPV were both performed in one procedure.

### Statistical analysis

The binary outcome was whether or not retinal re-detachment occurred. The predictor of interest was the type of retinopexy. Because the rate of re-detachment is known to depend on the presence of PVR and/or the type of tamponade (silicone oil, e.g. primary silicone oil in giant retinal tears), subgroup analyses were carried out to ensure that predictor-outcome associations were not confounded by these factors. In addition, adjusted analyses were carried out to account for patient characteristics (macula status). The association between the predictor (type of retinopexy) and the outcome (re-detachment) was analysed with a chi-squared test with continuity correction, either for all patients or for the high-risk subgroups (patients with primary PVR or with primary silicone oil tamponade). Generalized linear models with binomial error distribution were used to test whether predictor-outcome associations depend on PVR or the type of tamponade. Generalized linear models were also used for adjusted analyses of associations between predictors and outcome, taking patient characteristics (macula status) into account. A power calculation was performed to demonstrate that the study is adequately powered to detect a meaningful and expected difference in re-detachment rates between the two groups (the number of patients per group needed to show a significant difference with 90% probability was *n* = 342). All analyses were carried out with R, version 3.3.3.

## Results

Overall, 1017 consecutive eyes with RRD were included in this study. The median age at surgery was 63.2 years with a range between 15.2 and 94.5. Macula involvement occurred in 587 eyes (57.7%). Primary PVR was found in 93 eyes (9.1%) and silicone oil was used in 138 eyes (13.6%). In 492 eyes (48.4%) cryocoagulation and in 219 eyes (21.5%) endolaser only was performed. In 306 (30.1%) eyes both types of retinopexy were combined. The patient characteristics are shown in Table 1.

**Table 1:** Patients characteristics

Characteristics	Total	Cryo	Endolaser	Combined
	*n* = 1017 (%)	492 (48.4)	219 (21.5)	306 (30.1)
Sex				
Female	355 (34.9)	177 (17.4)	76 (7.5)	102 (10.0)
Male	662 (65.1)	315 (31.0)	143 (14.1)	204 (20.1)
Side				
Right	552 (54.3)	253 (24.9)	113 (11.1)	186 (18.2)
Left	465 (45.7)	239 (23.5)	106 (10.4)	120 (11.8)
Macula status				
On	430 (42.3)	189 (18.6)	83 (8.2)	158 (15.5)
Off	587 (57.7)	303 (29.8)	136 (13.4)	148 (14.6)
Lens status				
Phakic	501 (49.3)	232 (22.8)	101 (9.9)	168 (16.5)
Pseudophakic	516 (50.7)	260 (25.6)	118 (11.6)	138 (13.6)
Phacoemulsification	501 (49.3)	232 (22.8)	101 (9.9)	168 (16.5)
Primary PVR	93 (9.1)	35 (3.4)	39 (3.8)	19 (1.9)
Tamponade				
Gas	879 (86.4)	433 (42.6)	169 (16.6)	277 (27.2)
Silicone oil	138 (13.6)	59 (5.8)	50 (4.9)	29 (2.8)

The re-detachment rate was 10.1% (103 patients). Insufficiency of the retinopexy was the main reason for re-detachment, and occurred in 53.6% of these eyes. In 37.3% of patients, PVR-reaction led to re-detachment, and in 9.1% retinal detachment occurred at a different location caused by another break. In general, there was no significant difference in the re-detachment rate between the different types of retinopexy (*p* = 0.309). Further analysis showed that using cryotherapy is not associated with higher rates of re-detachments caused by PVR, or by insufficient treatment of retinal breaks when compared to endolaser alone (Table 2). Subgroup analysis showed a significantly higher rate of re-detachment in patients with a primary PVR (*p* = 0.0003), and in the group with silicone oil as the primary tamponade (*p* = 0.0001). The two confounders were strongly associated with each other (X^2^ = 290, *p* < 0.0001). Further, patients with a macula-off detachment experienced a significantly higher re-detachment rate (Table 3). Nevertheless, when associations between the type of retinopexy and re-detachment were separately analysed for the high-risk groups, the results were not different from those of all patients: again, there were no significant associations between predictors and outcome (Table 4).

**Table 2:** Associations between the occurrence of re-detachment and type of retinopexy, risk factors (primary PVR, silicon oil) and patient’s characteristics (macula status)

Predictor	Re-detachment			Chi-squared Test	
	No (n)	Yes (n)	Yes (%)	X^2^	*p*-value
Retinopexy					
Cryocoagulation	435	57	11.6	2.35	0.309
Endolaser	201	18	8.2		
Combined	278	28	9.2		
PVR					
No	841	83	9.0	13.21	0.0003
Yes	73	20	21.5		
Tamponade					
Gas	803	76	8.6	14.45	0.0001
Silicon oil	111	27	19.6		
Macula status					
On	397	33	7.7	4.47	0.034
Off	517	70	11.9

**Table 3:** Significance of association between retinopexy methods and cause of re-detachment

Retinopexy	Cause of re-detachment						Chi-squared Test	
	PVR		Insufficiency		New break			
	n	%	n	%	n	%	X^2^	*p*-value
Cryo	19	46	35	59	6	60	3.59	0.47
Endolaser	11	27	8	13	1	10		
Combined	11	27	16	28	3	30

**Table 4:** Association between the occurrence of re-detachment and type of retinopexy for high risk subgroups (with primary PVR or silicon oil tamponade)

	Re-detachment			Chi-squared Test	
	No (n)	Yes (n)	Yes (%)	X^2^	*p*-value
Primary PVR (*n* = 93) Retinopexy					
Cryocoagulation	27	8	22.9	0.59	0.744
Endolaser	32	7	18.0		
Combined	14	5	26.3		
Primary silicone oil (*n* = 138) Retinopexy					
Cryocoagulation	49	10	17.0	0.47	0.791
Endolaser	38	11	22.0		
Combined	23	6	20.7		

## Discussion

Creating a sufficient barrier around the retinal break is the crucial step when performing PPV for RRD, and one of the key factors for determining the outcome [4–6]. Usually, this is performed by using cryocoagulation and/or endolaser. Both methods for retinopexy cause an alteration of the blood-retinal barrier, which leads to a breakdown of the barrier and freeing RPE cells, which can cause PVR [13].

PVR is the clinical syndrome associated with retinal traction and detachment in which cells with proliferative potential multiply and contract on retinal surfaces. PVR is another important factor for surgical success after retinal detachment repair. Various studies report an incidence of PVR before surgery in 5–11% of all patients with RRD [3, 14, 15] and in 50–75% of re-detachment after retinal detachment repair PVR is the cause for failure [15]. In our study population, insufficiency of the retinopexy was the main reason for re-detachment, and occurred in 53.6% of patients, followed by PVR with 37.3%. In 9.1%, retinal re-detachment occurred at a different location caused by another break, which had newly developed or was missed during the first procedure. In this study, there was no significant difference in the re-detachment rate between the different types of retinopexy (*p* = 0.309). Further, cryotherapy was not associated with higher rates of re-detachments caused by PVR.

In both techniques, there is a reduction in adhesive strength in the first week caused by chorioretinal edema and cellular infiltration [6–8]. Until a sufficient adhesion is achieved, usually 2–3 weeks after surgery, a tamponade is required. Young et al. showed in an experimental study with rabbit retina that the adhesive force after photocoagulation was reduced 50% at 8 h, but increased beyond normal by 24 h, and remained twice normal between 3 days and 4 weeks [8]. The tamponade agents displace the preretinal and subretinal fluid away from retinal breaks and appose the retina in the underlying retinal pigment epithelium [16, 17]. Despite not reaching statistical significance, patients treated with cryotherapy had a tendency to have more insufficiency in the retinopexy and suffer from re-detachments caused by a new or missed break (Table 3). From our experience, most of the re-detachments caused by insufficiently treated breaks are not because of under- but rather due to overtreatment of the retinal break producing atrophic scars. A new type of retinopexy, such as high-frequency electric welding, shows promising results by creating an immediate chorioretinal adhesion, which could make the usage of tamponade obsolete [6].

The data suggests that the type of retinopexy has little relevance for the surgical outcome of PPV for the primary RRD. The only risk factors that contribute to a higher re-detachment rate in our study were the primary PVR and the primary use of silicone oil tamponade, and these two risk factors are highly associated with each other. Further, patients with macula off RRD have a higher risk for re-detachment, which probably due to the more extensive and longer persistent retinal detachment [18].

In conclusion, we recommend using the type of retinopexy you are more familiar with, as both techniques, if properly applied, have no major disadvantages in terms of higher re-detachment rate.

## Abbreviations

PPV: Pars plana vitrectomy
PVR: Proliferative vitreoretinopathy
RRD: Rhegmatogenous retinal detachment
